# Systematic evaluation of characteristics of the membrane-based fed-batch shake flask

**DOI:** 10.1186/s12934-017-0741-6

**Published:** 2017-07-17

**Authors:** P. Philip, K. Meier, D. Kern, J. Goldmanns, F. Stockmeier, C. Bähr, J. Büchs

**Affiliations:** AVT-Biochemical Engineering, RWTH Aachen University, Forckenbeckstraße 51, 52074 Aachen, Germany

**Keywords:** Fed-batch, Shake flask, Glucose release, Dialysis membrane, Microfiltration membrane, Small-scale screening tool, RAMOS, *Escherichia coli*

## Abstract

**Background:**

The initial part of process development involves extensive screening programs to identify optimal biological systems and cultivation conditions. For a successful scale-up, the operation mode on screening and production scale must be as close as possible. To enable screening under fed-batch conditions, the membrane-based fed-batch shake flask was developed. It is a shake flask mounted with a central feed reservoir with an integrated rotating membrane tip for a controlled substrate release. Building on the previously provided proof of principle for this tool, this work extends its application by constructive modifications and improved methodology to ensure reproducible performance.

**Results:**

The previously limited operation window was expanded by a systematic analysis of reservoir set-up variations for cultivations with the fast-growing organism *Escherichia coli*. Modifying the initial glucose concentration in the reservoir as well as interchanging the built-in membrane, resulted in glucose release rates and oxygen transfer rate levels during the fed-batch phase varying up to a factor of five. The range of utilizable membranes was extended from dialysis membranes to porous microfiltration membranes with the design of an appropriate membrane tip. The alteration of the membrane area, molecular weight cut-off and liquid volume in the reservoir offered additional parameters to fine-tune the duration of the initial batch phase, the oxygen transfer rate level of the fed-batch phase and the duration of feeding. It was shown that a homogeneous composition of the reservoir without a concentration gradient is ensured up to an initial glucose concentration of 750 g/L. Finally, the experimental validity of fed-batch shake flask cultivations was verified with comparable results obtained in a parallel fed-batch cultivation in a laboratory-scale stirred tank reactor.

**Conclusions:**

The membrane-based fed-batch shake flask is a reliable tool for small-scale screening under fed-batch conditions filling the gap between microtiter plates and scaled-down stirred tank reactors. The implemented reservoir system offers various set-up possibilities, which provide a wide range of process settings for diverse biological systems. As a screening tool, it accurately reflects the cultivation conditions in a fed-batch stirred tank reactor and enables a more efficient bioprocess development.

**Electronic supplementary material:**

The online version of this article (doi:10.1186/s12934-017-0741-6) contains supplementary material, which is available to authorized users.

## Background

Fed-batch cultivations have found extensive application in the field of biotechnology [1]. This operation mode is often used for the overexpression of biomolecules, such as nucleic acids [2], recombinant or natural proteins [3], amino acids [4] or the achievement of high cell densities [1]. The fed-batch operation mode is chosen for biological systems that evoke challenges as oxygen limitation, osmotic inhibition, catabolite repression, substrate inhibition or overflow metabolism when exposed to high initial substrate concentrations. This cultivation strategy has proven to be advantageous for a variety of microorganisms such as *Escherichia coli* [5], *Saccharomyces cerevisiae* [6], *Bacillus subtilis* [7] and *Pichia pastoris* [8]. Due to modern molecular evolutionary methods, the diversity of new strains and products has become innumerable. This trend calls for screening tools that help to identify suitable strains and process parameters for subsequent production processes [9] or downstream processing [10]. In the past, production processes were conducted in fed-batch mode, but the initial screening was conducted in batch mode. As physiological conditions and performance of strains between batch and fed-batch operation mode are not comparable, high-potential strains were often not identified [11–13]. Furthermore, the limited performance of sub-optimal strains had to be compensated with extensive process optimization, which was often not feasible. In worst case, the loss of a well-suited strain in the screening could not be compensated at all. The availability of fed-batch screening tools is an essential prerequisite for a fast-track implementation of production processes.

To conduct fed-batch screening experiments, a number of devices and materials are commercially available. The distinguishing factors are complexity of technical periphery, handling, option of integrated online analytics and process control. Before choosing a method, a comparison based on various evaluation criteria such as experimental throughput, variety of feedable compounds, possibility to adjust feed rates, necessity of pH control, etc. need to be taken into consideration [14]. Scaled-down small-scale stirred tank reactors from milliliter to liter scale enable the feeding of one or more required substances with the aid of pumps and control units (e.g. DASGIP, Jülich/Germany; INFORS HT, Bottmingen/Switzerland; etc.) [15, 16]. The pH can easily be controlled and the feeding-rate can be altered to any desired profile. The entire degree of freedom as in conventional stirred tank reactors is available and together with an online-monitoring system, the scale-up to large-scale stirred tank reactors can easily be conducted. However, due to the complexity of the required technical equipment, handling effort and investment costs are high and limit the experimental throughput. A similar functional system on a milliliter scale is the microfluidic microtiter plate system BioLector Pro (m2p-labs, Baesweiler/Germany). It offers the possibility to supply culture wells with substrates, pH stabilizing substances or any other compound from corresponding reservoir wells. The simultaneous cultivation of 32 microbioreactors is feasible [17, 18]. In combination with the BioLector, online-monitoring and process control are possible. For such a multi-functional performance, high investment and operation costs need to be procured. Alternatively, the EnBase (BioSilta Oy, Oulu/Finland) system is based on the enzymatic degradation of starch to glucose using glucoamylase from *Aspergillus niger.* This concept can be incorporated for various bioreactor scales [19–22]. Glucosidase is another enzymatic system used for the in situ glucose release by means of a soluble polymer [23]. The synthetic Feed in Time (m2p-labs, Baesweiler/Germany) fed-batch medium also provides glucose as carbon source [24]. The feeding rate can easily be altered by adapting the added enzyme concentration, enabling the possibility of high-throughput screening. Nevertheless, the significant impact of temperature and pH on enzyme activity has to be considered in cultivations. The shelf-life is strongly dependent on enzyme stability, and the application of this system is limited to amylase or protease-free cultivations. The FeedPlates and FeedBeads (Kühner, Birsfelden/Switzerland), applicable for microtiter plates and shake flasks, respectively are based on the diffusion-driven release of solid substrates encapsulated in a silicone matrix [25]. pH stabilization is possible through the release of sodium carbonate [26]. Even though the system is applicable in high-throughput, the possibility of adjusting the feed rate is limited to a variation of cultivation parameters, e.g. filling volume or by choosing a polymer material with a different release rate.

**Fig. 1:**
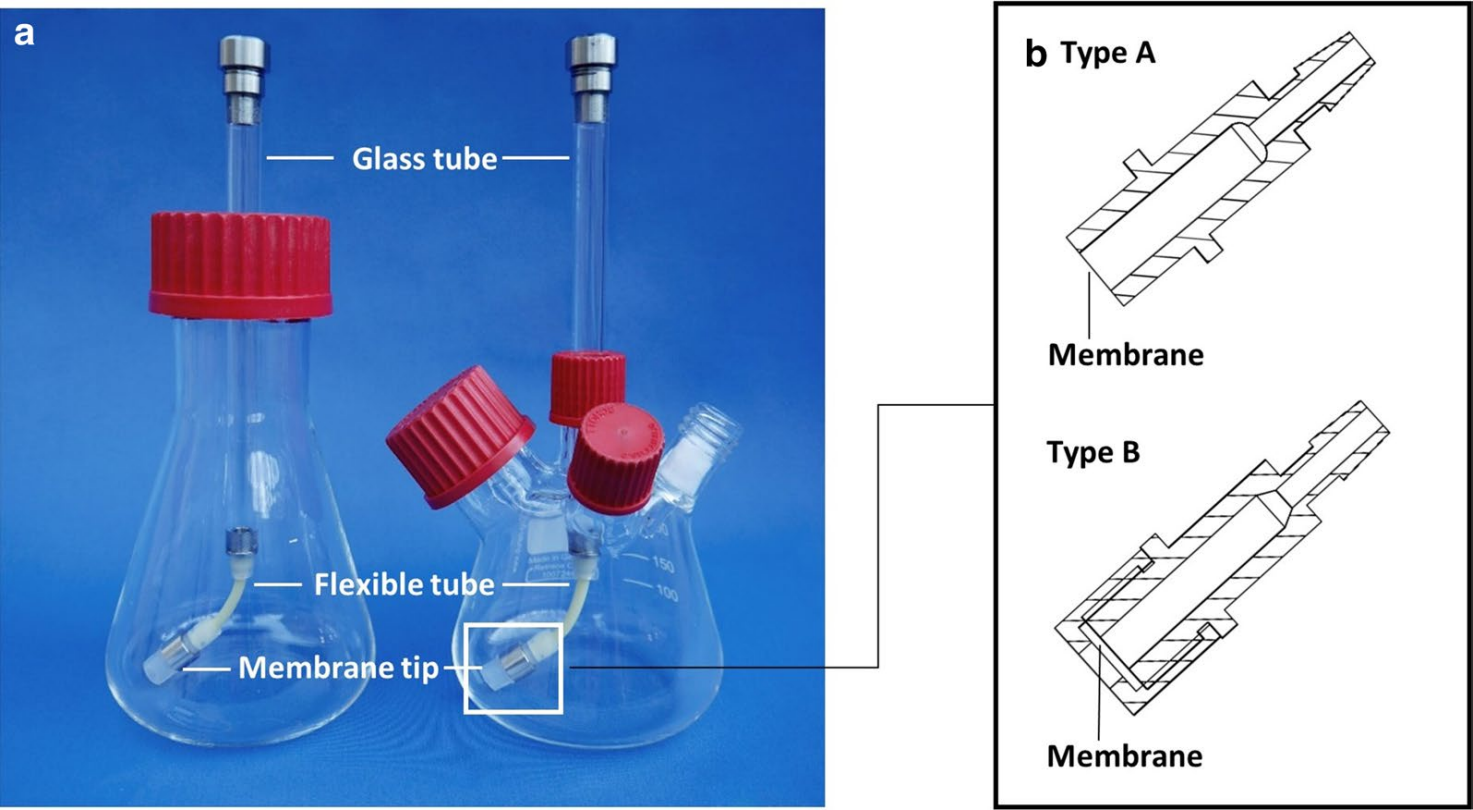
Set-up of the membrane-based fed-batch shake flask. **a** The offline (*left*) and the online (*right*) membrane-based fed-batch shake flasks have a reservoir system to enable the continuous supply of substrate to the culture broth. **b** Membrane tip type A requires an additional silicone ring, which spans the membrane on the tip; with the membrane tip type B the membrane is screwed between the cap and the body of the membrane tip

The prototype development of the membrane-based fed-batch shake flask conducted by Bähr et al. [14] aimed at designing a versatile yet cost-effective screening tool without external infrastructures such as pumps or control units. These technical simplifications enable a continual release of substrate mimicking a constant feeding profile. Yet, as shown by Bähr et al. [14] a stepwise increase in the feeding profile can be accomplished through feed supplementation with higher concentrated substrate solutions in the reservoir. The simplicity and robustness, reflected in the working principle and technical set-up, enable sufficient flexibility for parallelization. The controlled release of substrates is accomplished through a built-in rotating membrane tip. The larger culture volume in a shake flask, in comparison to microtiter plates, offers the possibility for higher sample volume necessary for offline analysis. Figure 1a (left) shows the offline version of the membrane-based fed-batch shake flask. The set-up is based on a central reservoir system consisting of three main parts mounted on a standard shake flask. The glass tube holds the dissolved components to be released, followed by a flexible tube allowing for an in-phase rotation of the membrane tip with the liquid bulk during shaking. The membrane tip enables the fixation of a membrane, the integral part responsible for the controlled release process. With the help of an in-house built apparatus, flexible membranes are everted on the membrane tip (Fig. 1b, type A) using a silicone ring to ensure a crease-free and consistent spanning of the membrane surface. No pressure-induced impact on the transport through the membrane needs to be considered, as the entire shake flask is placed under ambient pressure. Figure 1a (right) shows the online version of the flask. Additional parts for gas inlet and outlet enable the usage with the respiration activity monitoring system (RAMOS) [27]. This modification enables a quasi-continuous online monitoring of the respiration activity [oxygen transfer rate (OTR), carbon dioxide transfer rate (CTR), respiratory quotient (RQ)] and provides information on various metabolic phenomena, as well as limitations or inhibitions. The oxygen transfer rate (OTR) in a bioreactor can be assumed to be in a quasi-continuous state with the oxygen uptake (OUR) or consumption rate. It is a common parameter for the quantification of the physiological state of an aerobic culture [28]. Since most metabolic activities depend on oxygen consumption and are based on stoichiometric proportionalities, the OTR can be used as a parameter to evaluate the glucose-limited feeding of various fed-batch cultivations presented in this study. The maximum oxygen transfer capacity, referred to as OTR_max_, is the maximum gas–liquid mass transfer for a given set of operation conditions [29].

The proof of principle for this screening tool was provided by Bähr et al. [14] in cultivations with the controlled release of carbon and nitrogen sources. In this work, the prototype was constructively modified and improved to ensure a more reproducible performance. The previously limited operation window was expanded by the systematic analysis of reservoir set-up variations for cultivations with the fast-growing organism *E. coli* and provides the basis for a quick adaptation to further biological systems. In a final step, the application of the membrane-based fed-batch shake flask as a screening tool was validated in a parallel experiment with a laboratory-scale stirred tank reactor.

## Results and discussion

### Concentration-related issues with the membrane-based fed-batch shake flask

**Fig. 2:**
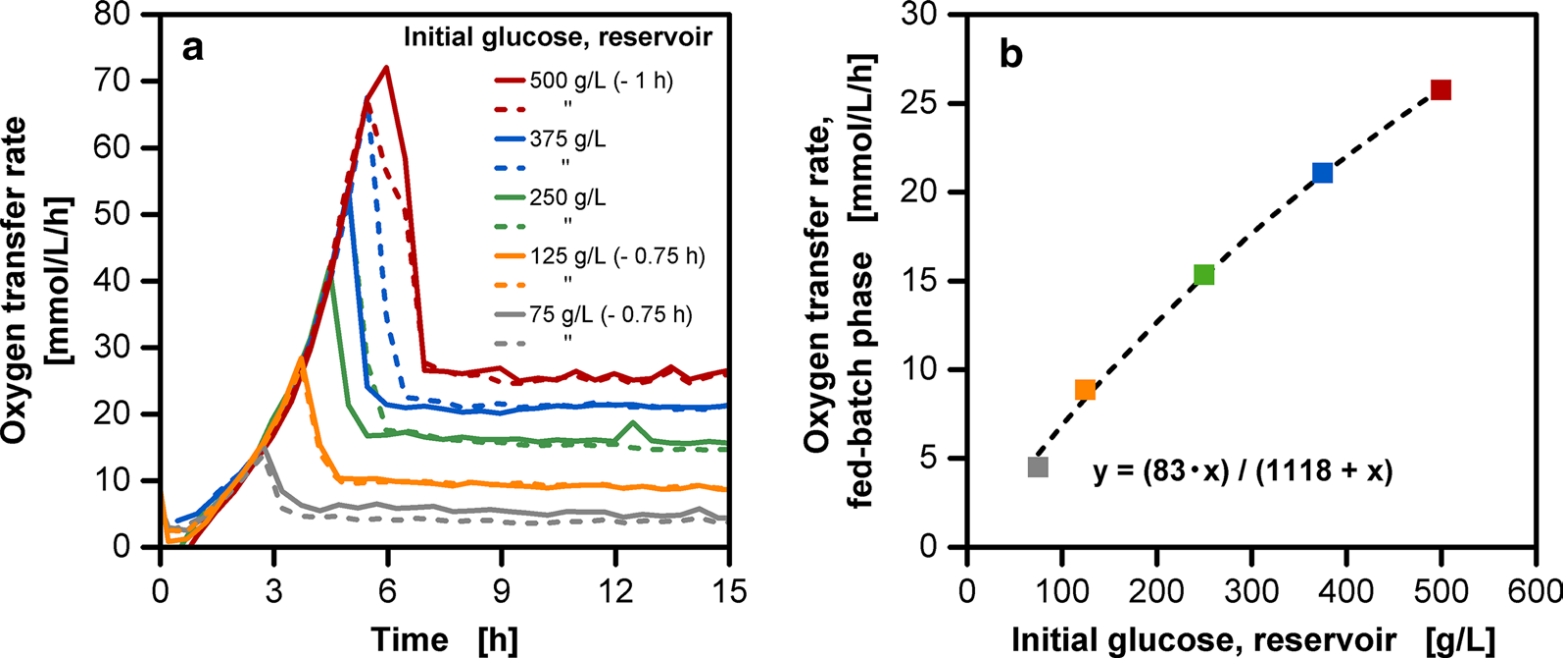
Effect of initial reservoir glucose concentration on oxygen transfer rate of *E. coli* fed-batch cultivations (duplicates). **a** For clarity reasons the cultivations with 75, 125 and 500 g/L initial reservoir glucose concentration have been shifted timewise as indicated, due to deviating lag-phases. **b** The oxygen transfer rate level of the fed-batch phase is respectively plotted over the initial reservoir glucose concentration; cultivation conditions: Wilms-MOPS-mineral medium (0.2 M MOPS), temperature: 37 °C, shaking frequency: 350 rpm, shaking diameter: 50 mm, culture broth volume: 10 mL, inoculation OD_600_: 0.5, reservoir filling volume: 2 mL, dialysis membrane: Reichelt (1) dialysis membrane, membrane area: 18.1 mm^2^; strain: *E. coli* BL21 (DE3) pRhotHi-2-EcFbFP

A controlled supply of substrate over the cultivation time enables to regulate the metabolic activity of a biological system within a desired corridor. One option to accomplish this is to alter the initial substrate concentration in the feed reservoir. In Fig. 2a oxygen transfer rate (OTR) is shown over cultivation time for several initial glucose concentrations in the reservoir. The Reichelt (1) dialysis membrane used in the following experimental study is permeable to low molecular weight solutes as glucose and ions but impermeable to high molecular weight proteins [30]. The mechanistic principle for substrate release is based on diffusion. No glucose was provided in the initial composition of the culture medium in all the fed-batch cultivations of this study. Initially low biomass concentrations are present in the culture medium. In this phase, also termed as batch phase, the substrate demand is lower than the substrate supply from the reservoir, resulting in an initial accumulation of glucose [14, 25]. With increasing biomass concentrations, the accumulated glucose is getting consumed until it becomes limited resulting in a fed-batch phase. As seen in Fig. 2, the extensive variation of the initial glucose accumulation does not cause a prolonged period of oxygen limitation in cultivations, which is a common cause for alterations of an organism’s metabolism [31]. Overflow metabolism, in the form of acetate production, was not detectable in both RAMOS as well as high-performance liquid chromatography (HPLC) measurements (data not shown). With increasing initial concentration in the reservoir, a higher amount of released glucose results in both higher OTR maxima during the batch phase varying up to a factor of seven and higher OTR levels in the fed-batch phase varying up to a factor of five.

**Fig. 3:**
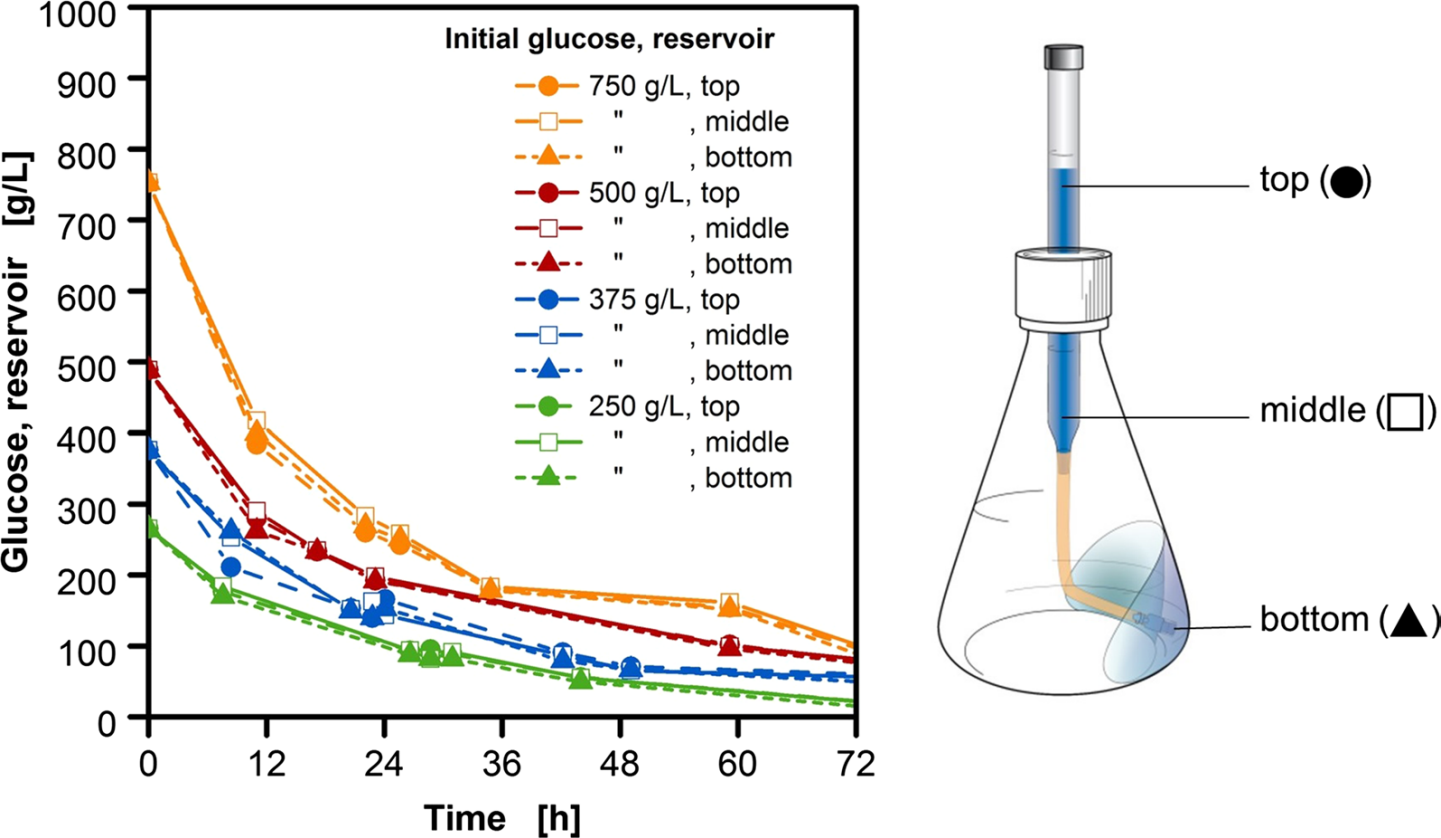
Analysis of glucose concentrations at three different positions in the reservoir system in *E. coli* fed-batch cultivations. Samples were taken at the upper liquid surface (top, *filled circles*), from the middle of the glass tube (middle, *open squares*) and behind the membrane (bottom, *filled triangles*) during cultivations for varied initial reservoir glucose concentrations (250, 375, 500 and 750 g/L); cultivation conditions: Wilms-MOPS-mineral medium (0.2 M MOPS), temperature: 37 °C, shaking frequency: 350 rpm, shaking diameter: 50 mm, culture broth volume: 10 mL, inoculation OD_600_: 0.5, reservoir filling volume: 2 mL, dialysis membrane: Reichelt (1) dialysis membrane, membrane area: 18.1 mm^2^; strain: *E. coli* BL21 (DE3) pRhotHi-2-EcFbFP

The correlation between initial glucose concentration in the reservoir and OTR level in the fed-batch phase is hyperbolic (Fig. 2b). Bähr et al. [14] describe that the glucose concentration gradient between the culture medium and the reservoir causes water to diffuse into the feed reservoir. This leads to a dilution of the feed and a decreased glucose gradient. A non-linear decrease of the concentration profile can be observed over time in the reservoir (Fig. 3) contributing to the above-mentioned effect. In addition, diffusion coefficients for glucose solutions decrease linearly with increasing concentration, having an asymptotic progress at high concentrations [32]. Several studies with alternative membranes have shown a similar hyperbolic correlation to a substrate transport. Paul et al. [33] propose that the viscosity of the solvent penetrating through the membrane may be the underlying cause for the non-linear transport. Almazán et al. [34] suggest that concentration polarization and rise of osmotic pressure are the causes for the decline of flux through the membrane due to increased resistance to permeation. Further, it was suggested that glucose as an uncharged organic molecule, but with a high dipole moment (~14 Debye) is more likely to be repelled by a membrane charge [35]. Further, the dialysis membrane in this experimental set-up is placed in a biological surrounding with a complex media composition. Even though regenerated cellulose exhibits low protein binding characteristics [36], the contact with organic and non-organic compounds could lead in time to the occurrence of fouling. The cultivation results with the cellulose-based dialysis membrane show that despite possible complexities resulting from solvent–solute-membrane interaction, a reproducible glucose release can be accomplished. The correlation presented in Fig. 2b enables a quick and simple adaptation of the initial glucose concentration to the demands of both slow and fast-growing microorganisms.

A homogenous reservoir composition without a concentration gradient is essential to provide the basis for a comparable substrate release for the membrane-based fed-batch shake flask. To address the question whether a concentration gradient is prevalent along the reservoir height, samples for HPLC analysis were taken at the upper liquid surface, the middle portion of the glass tube and directly behind the membrane by piercing through the membrane from the front (Fig. 3). In all four experiments with different initial glucose concentration in the reservoir, even for the highest initial concentration of 750 g/L glucose in the reservoir, no significant deviation in glucose concentrations, measured at different positions, could be determined. The detected slight deviation results primarily from errors due to the high dilution factors necessary for HPLC analysis. To achieve the amount of released glucose based on Fick’s law, a rough calculation of the theoretical necessary concentration gradient was conducted [data not shown]. To accomplish the release of glucose solely based on diffusion, an unrealistic high concentration gradient would have been necessary. This confirms the existence of convective transport and good mixing within the reservoir through vigorous shaking of 350 rpm at 50 mm shaking diameter. These settings were chosen to supply sufficient oxygen to fast-growing *E. coli* cultivations. For lower shaking frequencies for different experimental set-ups, this issue needs to be evaluated again.

**Fig. 4:**
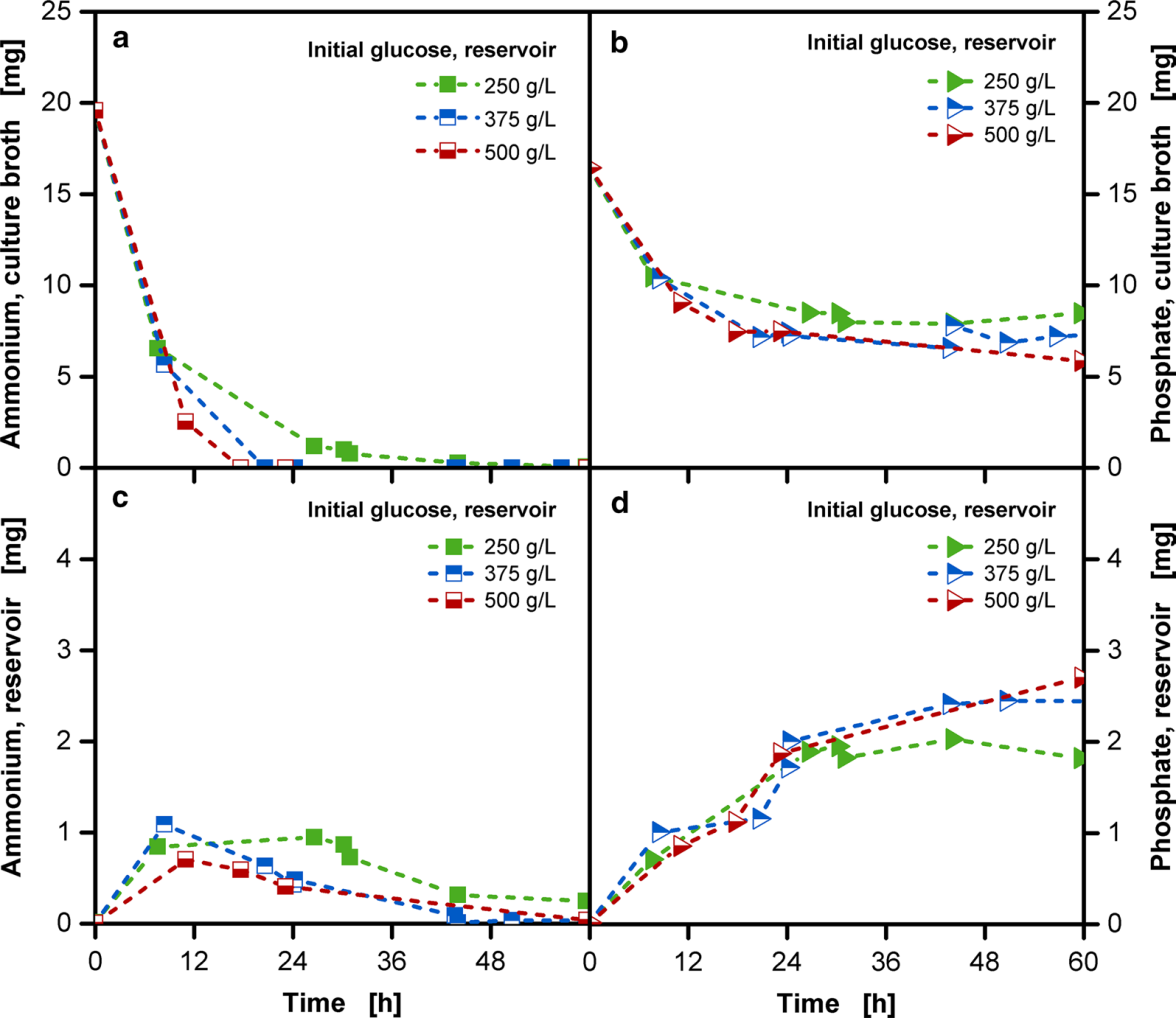
Distribution of ammonium and phosphate in the culture broth and the reservoir over time in *E. coli* fed-batch cultivations. **a**, **c** Ammonium amount for three initial reservoir glucose concentrations (250, 375 and 500 g/L). **b**, **d** Phosphate amount for three initial reservoir glucose concentrations (250, 375 and 500 g/L); cultivation conditions: Wilms-MOPS-mineral medium (0.2 M MOPS), temperature: 37 °C, shaking frequency: 350 rpm, shaking diameter: 50 mm, culture broth volume: 10 mL, inoculation OD_600_: 0.5, reservoir filling volume: 2 mL, dialysis membrane: Reichelt (1) dialysis membrane, membrane area: 18.1 mm^2^; strain: *E. coli* BL21 (DE3) pRhotHi-2-EcFbFP

To address the question whether essential nutrients diffuse in the opposite direction from the culture medium into the reservoir and are lost to the cultivation, measurements of ammonium and phosphate in both the culture broth and the reservoir were conducted. Figure 4a, c show for ammonium an initial accumulation in the reservoir in all tested cultivations. Over the time, ammonium is consumed and once the concentration is decreased in the culture broth, a back-diffusion of ammonium from the reservoir takes place. Hence, the microorganisms can completely metabolize the initially provided amount of ammonium. For cultivations with an initial glucose concentration in the reservoir of 375 and 500 g/L an ammonium limitation can be observed after approximately 18 and 21 h, respectively. The effect of nitrogen limitation on a cultivation along with the means to overcome this challenge is a crucial issue that needs to be addressed to conduct cultivations optimally. For phosphate, Fig. 4b, d show that the high amount provided initially to the culture broth results in an almost constant level for both the reservoir and the culture broth. As phosphate was not consumed in significant amounts no limitation in the culture broth occurred. In summary, the composition of the culture broth and the reservoir can result in a diffusive transport of media components into the reservoir. But the reservoir does not act as a permanent nutrient sink as back-diffusion to the culture broth can take place upon lowered concentration of the component in the culture broth.

### Additional set-up variations of the reservoir system

**Fig. 5:**
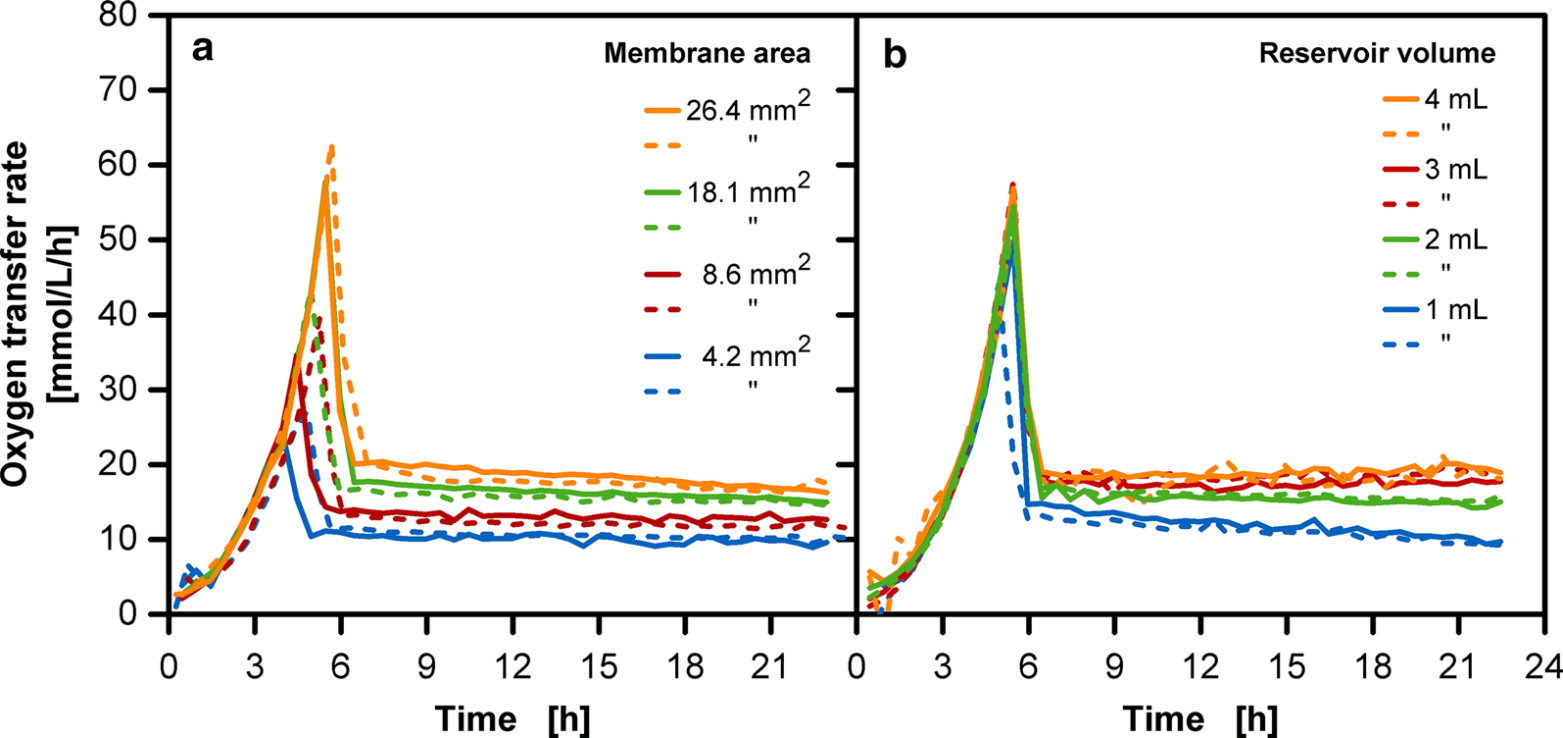
Effects of membrane area and reservoir volume on oxygen transfer rate in *E. coli* fed-batch cultivations (duplicates). Variation of **a** membrane area. **b** Reservoir volume; cultivation conditions: Wilms-MOPS-mineral medium (0.2 M MOPS, 250 g/L glucose in the reservoir), temperature: 37 °C, shaking frequency: 350 rpm, shaking diameter: 50 mm, culture broth volume: 10 mL, inoculation OD_600_: 0.5, 2 mL reservoir filling volume in **a** and varied reservoir filling volume in **b**, dialysis membranes: Reichelt (1) dialysis membrane, varied membrane area in **a** and 18.1 mm^2^ membrane area in **b**; strain: *E. coli* BL21 (DE3) pRhotHi-2-EcFbFP

In addition to the variation of the initial glucose concentration in the reservoir, the set-up of the reservoir system offers further possibilities to alter the release rate. One possibility is to alter the membrane area at the membrane tip (Fig. 5a). Increasing the membrane area from 4.2 to 26.4 mm^2^ has a direct influence on the peak height of the OTR in the batch phase, increasing from approximately 20 to 60 mmol/L/h. An OTR variation of about 10 mmol/L/h in the fed-batch phase can be observed between the lowest and the highest membrane area. Even though the membrane area was increased by a factor of almost six, this magnitude of alteration is not transferred to the OTR in the fed-batch phase. As the same initial glucose concentration is used for all set-ups, the increased batch peaks for larger membrane areas result in a higher amount of metabolized glucose at an early stage of cultivation. This may leave a reduced amount of glucose available in the reservoir for the fed-batch phase. The lower concentration gradient over the membranes results in a reduced driving force for glucose transport and hence, a lower OTR level of the fed-batch phase. Under the given conditions the alteration of the membrane area allows for a fine-tuning of the release rate. A simple way to reduce the height and width of the batch peak is through increasing the inoculation concentration (see Additional file 1). Cultivations reach the glucose-limited condition faster and more glucose is available for the fed-batch phase.

The duration of the fed-batch phase at a more or less constant OTR level can be controlled via the reservoir filling volume (Fig. 5b). The initial concentration difference between the reservoir and the culture broth remains the same, but due to the higher total amount of glucose, the concentration gradient for diffusion is maintained for a longer period of time. For long-term cultivations, a higher reservoir volume is advantageous for the longer benefit from the fed-batch phase. In addition, the influence of membrane thickness was tested for a 28 and 42 µm thick dialysis membrane (see Additional file 2). Astonishingly, no difference in the release rate could be identified for these two types of membranes.

**Fig. 6:**
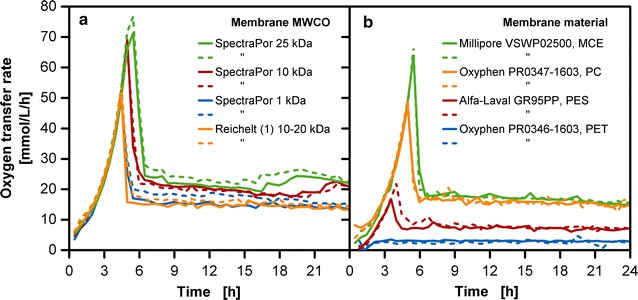
Effect of molecular weight cut-off (MWCO), membrane type and material on oxygen transfer rate in *E. coli* fed-batch cultivations (duplicates). **a** Membrane tip type A (see Fig. 1b) with membrane area of 18.1 mm^2^ was used for the dialysis membranes with varying MWCO values [SpectraPor 25 kDa, SpectraPor 10 kDa, SpectraPor 1 kDa, Reichelt (1) dialysis membrane]. **b** Membrane tip type B (see Fig. 1b) with membrane area of 11.34 mm^2^ was used for membranes of varying materials (mixed cellulose ester (MCE), polycarbonate (PC), polyether sulfone (PES), polyethylene terephthalate (PET)); cultivation conditions: Wilms-MOPS-mineral medium (0.2 M MOPS, 250 g/L glucose in the reservoir), temperature: 37 °C, shaking frequency: 350 rpm, shaking diameter: 50 mm, culture broth volume: 10 mL, inoculation OD_600_: 0.5, reservoir filling volume: 2 mL; strain: *E. coli* BL21 (DE3) pRhotHi-2-EcFbFP

Figure 6a shows a selection of membranes that are sufficiently flexible to be used with the membrane tip type A (see Fig. 1b). In contrast, Fig. 6b shows membranes which are stiff and have a paper-like nature. For these, the membrane tip type B (Fig. 1b) was used. With the usage of both membrane tips the optional range of utilizable membranes was extended. Figure 6a shows the Reichelt (1) dialysis membrane (10–20 kDa MWCO) in comparison with membranes from SpectraPor with a defined MWCO. With 180 Da, glucose is many times smaller than the MWCO of the tested membranes and no rejection effects should occur. Yet with a change of the MWCO the possibility of fine-tuning the release rate can be observed. For dialysis membranes, it is postulated that diffusion through the membrane is dependent on the amount of water present in the wet membrane [30], which most likely varies in the tested membranes. The cultivation with the Reichelt (1) dialysis membrane is similar to the cultivation with the 1 kDa MWCO membrane from SpectraPor. This discrepancy may result from the fact that no standardization is available for the tested solute or the operation conditions for MWCO measurements [37]. An increase in the batch peak and OTR level of the fed-batch phase can be detected for increasing MWCOs of the SpectraPor membranes. Even though an initial concentration of 250 g/L was used in the reservoir, the performance of SpectraPor 10 and SpectraPor 25 is similar to the cultivation with 375 g/L initial reservoir glucose concentration in combination with the Reichelt (1) dialysis membrane (see Fig. 2).

In addition to cellulosic dialysis membranes, alternative membranes are an asset for the application in the membrane-based fed-batch shake flask. A fundamental reason that may dictate the choice of alternative membrane materials to the cellulose-based dialysis membrane is the implementation in cellulase-containing systems. Especially, fermentation processes revolving around bio-fuel production may contain a cocktail of cellulases [38, 39]. Cellulases can be produced in fed-batch operation mode to achieve both a high titer and volumetric productivity [40]. For these applications, alternative membranes not based on cellulose are required for screening with the membrane-based fed-batch shake flask.

Figure 6b shows results obtained with membranes of different materials (Table 1). The selected membrane materials were tested for biocompatibility (data not shown) as described by Meier et al. [41]. In Fig. 6b, OTR values at various levels can be observed for identical initial settings. No distinction between batch and fed-batch phase can be seen with the Oxyphen PR0346-1603 membrane. It has a very low release rate for glucose and is appropriate for slow-growing organisms, such as plant cell cultivations [42]. For moderately fast-growing microorganisms e.g. yeast cultivations, the Alfa-Laval GR95PP membrane is more suitable. The moderate glucose release rate is likely to result from the membrane material PES, which has a negative membrane charge in an alkaline to neutral pH environment [43]. The intensity of a charged surface towards surrounding particles is strongly dependent on the electrostatic shield resulting from the counter-ions. As the media contains a number of salts and the pH is in the range of about 7–7.5, an interaction of ions with the membrane is very likely. The Millipore VSWP02500 has a similar performance as the previously discussed Reichelt (1) dialysis membrane and is based on a cellulose mixture. A dominant batch peak at about 70 mmol/L/h and an OTR level of 20 mmol/L/h in the fed-batch phase can be seen for an initial glucose concentration of 250 g/L in the reservoir. Similar characteristics are observed for the Oxyphen PR0347-1603 made of polycarbonate. These high glucose releases are suitable for fast-growing microorganisms, such as *E. coli*.

**Table 1:** Overview of tested membranes

Manufacturer	Membrane	MWCO/pore size	Material
Reichelt (1) (RCT Reichelt Chemietechnik GmbH + Co., Heidelberg/Germany)	RCT-NatureFlex-NP	10–20 kDa, 42 µm membrane thickness	Cellulose
Reichelt (2) (RCT Reichelt Chemietechnik GmbH + Co., Heidelberg/Germany)	RCT-NatureFlex-NP	10–20 kDa, 28 µm membrane thickness	Cellulose
SpectraPor (Spectrum Europe B.V., Breda/Netherlands)	Spectra/Por 7	1 kDa	Cellulose
SpectraPor (Spectrum Europe B.V., Breda/Netherlands)	Spectra/Por 7	10 kDa	Cellulose
SpectraPor (Spectrum Europe B.V., Breda/Netherlands)	Spectra/Por 7	25 kDa	Cellulose
Merck Millipore (Merck KGaA, Darmstadt/Germany)	VCTP Isopore	0.1 µm	Polycarbonate (PC)
Merck Millipore (Merck KGaA, Darmstadt/Germany)	VSWP02500 MF-Millipore	0.025 µm	Mixed cellulose ester (MCE)
Alfa-Laval (Alfa Laval Mid Europe GmbH, Glinde/Germany)	GR95PP	2 kDa	Polyether sulfone (PES)
Oxyphen (Oxyphen AG, Wetzikon/Switzerland)	PR0346-1603	0.03 µm	Polyethylene terephthalate (PET)
Oxyphen (Oxyphen AG, Wetzikon/Switzerland)	PR0347-1603	0.1 µm	Polycarbonate (PC)

### Comparative cultivation studies with a dialysis membrane and a microfiltration membrane

**Fig. 7:**
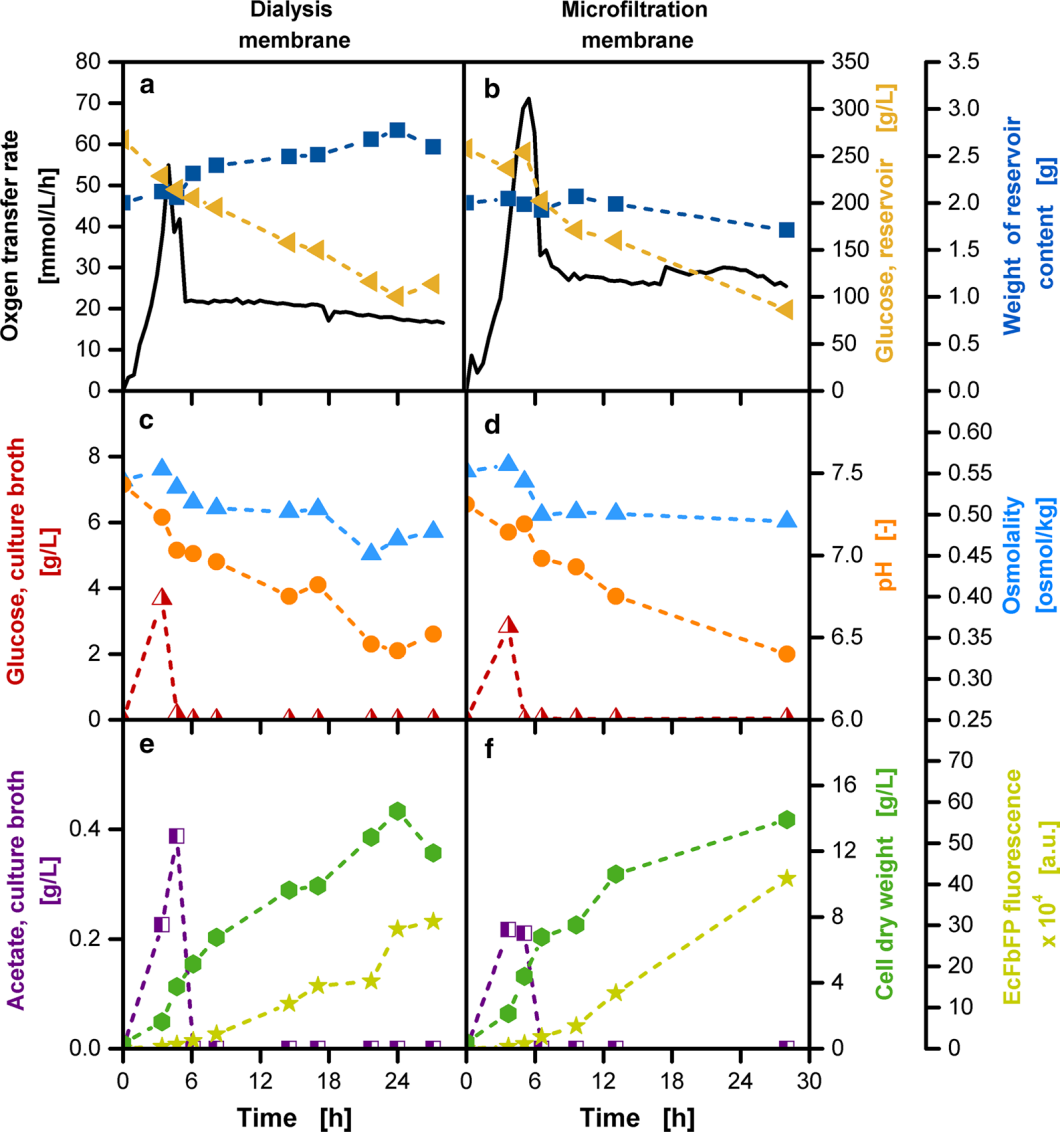
Online and offline characterization of cultivations with a dialysis membrane and a microfiltration membrane in *E. coli* fed-batch cultivations. **a**, **b** Oxygen transfer rate (OTR, *solid line*), glucose concentration in the reservoir (*slanted triangles*), glucose concentration in the culture broth (*straight triangles*), weight of reservoir content (*filled squares*). **c**, **d** Glucose concentration in the culture broth (*half-filled triangles*), pH (*circles*), osmolality (*filled triangles*) in the culture broth. **e**, **f** Acetate concentration (*half-filled squares*), cell dry weight (*hexagons*) and EcFbFP fluorescence (*stars*); cultivation conditions: Wilms-MOPS-mineral medium (0.2 M MOPS, 250 g/L glucose in the reservoir), temperature: 37 °C, shaking frequency: 350 rpm, shaking diameter: 50 mm, culture broth volume: 10 mL, inoculation OD_600_: 0.7, reservoir filling volume: 2 mL, dialysis membrane: Reichelt (1) dialysis membrane, membrane area: 18.1 mm^2^, microfiltration membrane: Millipore VCTP Isopore, membrane area: 11.34 mm^2^; strain: *E. coli* BL21 (DE3) pRhotHi-2-EcFbFP

The choice of the membrane material and type are important parameters to vary the substrate release from the reservoir. In Fig. 7 the usage of a dialysis membrane (Reichelt (1), 10–20 kDa) and a microfiltration membrane (Millipore VCTP Isopore, 0.1 µm) are compared to analyze the effects of varying transport mechanisms on the cultivation progress. The microfiltration membrane from Millipore VCTP Isopore is a track-etched membrane with a narrow pore size distribution, which is a unique property for membranes of this manufacturing process [44]. The pores are described to be circular holes with a sinusoidal wall variation [45].

Diffusive effects dominate the material flows within dialysis membranes, whereas hydrodynamic effects play a role in microfiltration membranes. With the dialysis membrane, an increase in the total amount of fluid weight in the reservoir over the cultivation time can be detected (Fig. 7a). Based on diffusive effects a compensation of a high glucose concentration in the reservoir and a high water content in the culture broth causes the flow of both substances to the respective other side [46]. With the microfiltration membrane, the transport through the membrane lattice in addition to the flow through distinct pores needs to be considered [47]. The effect of mass transport through diffusive processes is apparent in the non-constant progress of the glucose reservoir concentration and the initial constant phase of the reservoir fluid content (Fig. 7b). With declining glucose concentration, the impact of diffusion diminishes and the effects of convective flow are apparent with the decreasing amount of fluid in the reservoir.

In preliminary experiments, a faster glucose release was identified for the microfiltration membrane. To adapt to the glucose release with the dialysis membrane, a smaller membrane area was chosen. Figure 7a, b show the OTR results for both cultivations. Slightly higher OTR values can be observed for the batch and the fed-batch phase of the microfiltration membrane in comparison to the dialysis membrane. Despite these differences, comparable offline results were obtained for the preset settings. The values for osmolality (Fig. 7c, d) are in a similar range for both membrane types throughout the entire cultivation time. After an initial minimal increase, caused by glucose accumulation (Fig. 7c, d) and acetate production (Fig. 7e, f), the osmolality is reduced as the biomass increases, metabolizing the initially provided amount of nutrients. A horizontal progress of the osmolality is observed for most parts of the fed-batch phase, which results from the controlled release of the substrate and its immediate metabolism. The overall high value for osmolality results from high buffer concentrations necessary to stabilize the pH value in shake flask cultivations. An indication for the ongoing metabolism is the decreasing pH, which results from acidification by acetate and ammonium uptake. For both membrane types, similar values for offline measurements of pH, glucose and acetate concentration in the culture broth (Fig. 7c–f) were obtained. Figure 7e, f contain values for cell dry weight (CDW) after correction to avoid falsification due to concentration or dilution effects. The correction factor is based on the ratio between the initial and current flask content. An exponential increase of biomass can be noted in the batch phase until the accumulated glucose is exhausted. In the fed-batch phase, the self-regulatory feature of the system becomes apparent in that the microorganisms display linear growth. *E. coli* BL21 (DE3) pRhotHi-2-FbFP produces a flavine mononucleotide binding fluorescence protein (EcFbFP) as a product. For both membranes, low values of product are measured in the batch phase in the presence of an excess amount of glucose. After entering the glucose-limited fed-batch phase an almost tenfold increase in the product concentration along with a doubled biomass amount is noted until the end of the given cultivation time. This is a typical feature of a catabolite-repressed system [14]. Despite the different transport mechanisms of the two membranes a similar performance can be obtained for the same initial glucose concentration by adapting the membrane area.

### Parallel cultivations in shake flasks and laboratory-scale stirred tank reactors in batch and fed-batch operation mode

**Fig. 8:**
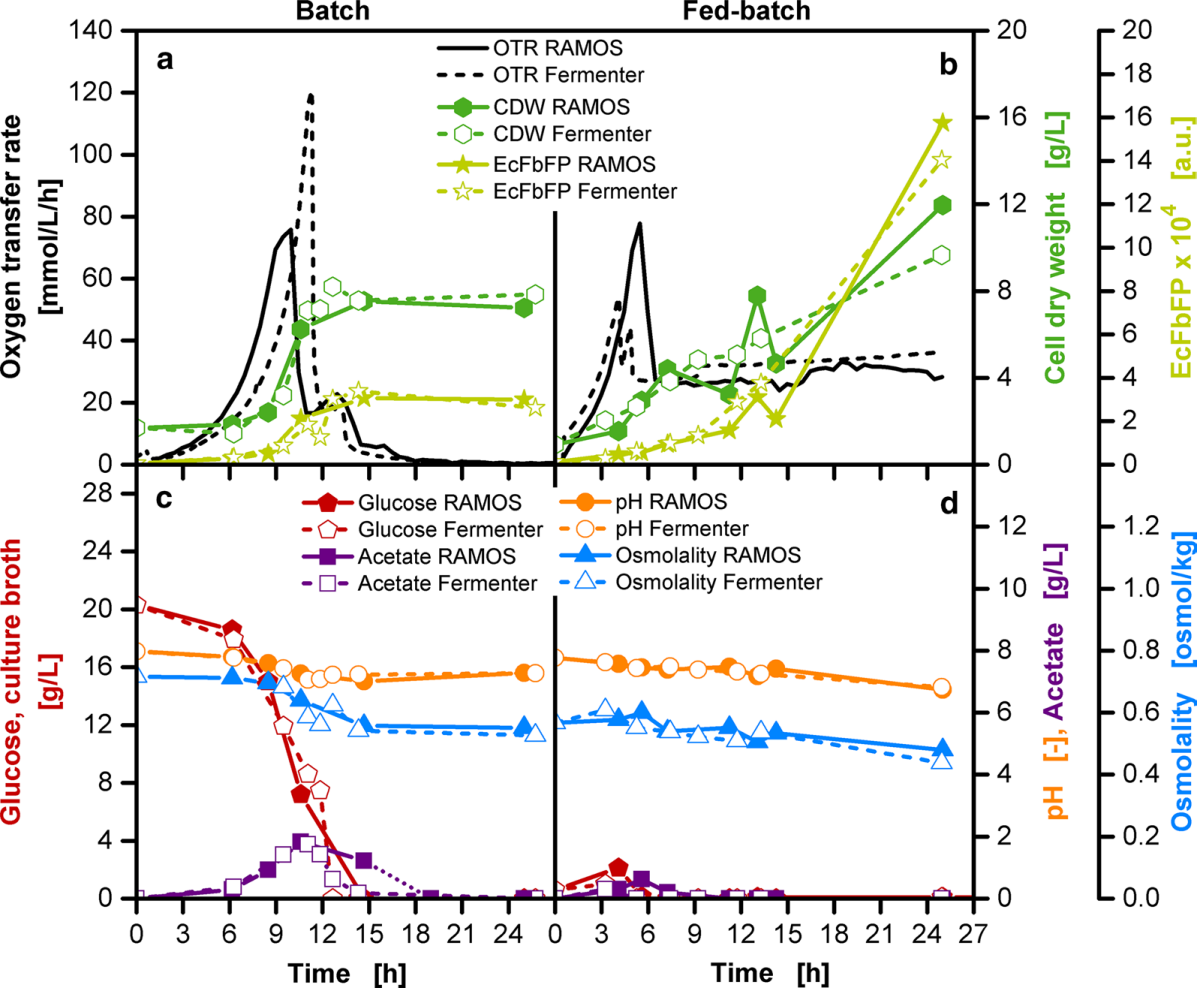
Parallel cultivations in shake flasks and laboratory-scale stirred tank reactors in batch and fed-batch operation mode with *E. coli*. **a**, **c** Batch cultivations with Wilms-MOPS-mineral medium (0.2 M MOPS), 20 g/L glucose, inoculation OD_600_: 0.8. **b**, **d** Fed-batch cultivations with Wilms-MOPS-mineral medium (0.2 M MOPS), without initially provided glucose in culture broth, glucose in the reservoir of the fed-batch shake flask and in the feed bottle of the stirred tank reactor: 250 g/L, feeding of the fed-batch stirred tank reactor is started at the beginning of the experiment with a pumping rate of 4.2 mL/h, inoculation OD_600_: 0.9; shake flask cultivation conditions: temperature: 37 °C, shaking frequency: 350 rpm, shaking diameter: 50 mm, culture broth volume: 10 mL, fed-batch reservoir filling volume: 2 mL, microfiltration membrane: Merck Millipore Isopore VCTP, membrane area: 11.34 mm^2^; stirred tank reactor conditions: temperature: 37 °C, aeration: 1 vvm, DOT controlled at 30% air saturation, reactor working volume: 1 L; strain: *E. coli* BL21 (DE3) pRhotHi-2-EcFbFP

The primary aim of this work was to introduce the potentiality of the fed-batch shake flask fulfilling diverse requirements of fed-batch applications. A screening tool is only good when it accurately reflects the cultivation conditions of stirred tank reactors with higher volumes. To validate the functionality of the membrane-based fed-batch shake flask, parallel cultivations in a RAMOS device and in a laboratory-scale stirred tank reactor with a working volume of 1 L were conducted. The microfiltration membrane (Millipore VCTP Isopore, 0.1 µm) introduced in Fig. 7 was used with the membrane tip type B (Fig. 1b). To estimate the released amount of glucose over time, the glucose concentration in the reservoir and the weight of the reservoir content have to be determined in advance (Fig. 7b). With the help of a simplified density calculation [48] the volume of the reservoir content was calculated. Approximately 0.013 g/h of glucose has been provided to 10 mL culture broth of a membrane-based fed-batch shake flask. An equivalent glucose release rate was implemented with an appropriate pump setting for a 1 L laboratory-scale stirred tank reactor with a 250 g/L glucose feed stock. It is to be noted that the common practice of providing an initial amount of glucose in the culture broth and subsequently starting the glucose feed with a pump was not followed in this experimental set-up. In the membrane-based fed-batch shake flask no glucose is provided to the initial culture broth, as the glucose release from the reservoir starts immediately at the beginning of the experiment. The same procedure was followed for the laboratory-scale stirred tank reactor. No glucose was provided to the initial culture broth and the glucose supply was started with the help of a pump directly at the start of an experiment. The total volume of the 250 g/L feed solution pumped into the stirred tank reactor amounts to 110.9 mL, equivalent to 27.7 g glucose in 25 h. For comparative reasons, the same experimental procedure was carried out on both scales for the batch operation mode with 20 g/L glucose initially provided in the culture medium. The slight time shifts in Fig. 8 result from the consecutive start of the various devices used.

Figure 8a shows the OTR for the RAMOS and laboratory-scale stirred tank reactor cultivations in the batch operation mode. The difference in the initial OTR peak height is most probably due to a short period of oxygen limitation in shake flask cultivations. For the attempted comparison, it can be regarded as irrelevant, as offline samples reveal a similar performance on both scales (Fig. 8a, c). The initial OTR peak results from the metabolism of glucose as seen in the decreasing glucose concentration initially provided to the culture medium (Fig. 8c). An exponential increase in biomass can be noted for this phase. Due to overflow metabolism, acetate is being produced in parallel (Fig. 8c) [49–51]. After depletion of glucose, acetate is consumed and results in a second OTR peak [52]. A minimal increase in biomass can be noted up to a final constant level until the end of the cultivation. Upon depletion of glucose, an increase in product concentration (EcFbFP) can be observed, reaching a constant level until the end of the cultivation. Throughout the cultivation, a decrease in osmolality and pH was detectable.

Although a slightly higher OTR level in the fed-batch phase is observable in the fed-batch stirred tank reactor compared to the shake flask cultivation (Fig. 8b), there is a good consistency between the offline data acquired with both systems (Fig. 8b, d). An initial glucose accumulation between 1 and 2 g/L can be noted in the initial batch phase (Fig. 8d). The glucose release from the fed-batch screening device adequately represents the glucose release via a pump to a fed-batch stirred tank reactor.

In the batch cultivations, the initial concentration of substrate increases the osmolality in the culture medium to 0.72 osmol/kg, in comparison to 0.57 osmol/kg in the fed-batch cultivations (Fig. 8c, d). This slight increase already had a significant impact on the lag-phase, which is almost doubled in the batch cultivation in contrast to the fed-batch cultivation. Even though a higher amount of total glucose was consumed in fed-batch operation mode than in batch operation mode, a lower acetate production is to be noted (Fig. 8c, d). 1.6–1.8 g/L of acetate was measured in the batch cultivation in comparison to 0.3–0.6 g/L in the fed-batch operation mode. A pH decrease as a result of acidification due to ammonium uptake is in a similar range for both shake flask and stirred tank reactor cultivations.

Comparison of Fig. 8a, b shows that the amount of biomass is increased by one-third in the fed-batch cultivation as a direct consequence of a higher amount of substrate provided. Due to the de-repressed state in fed-batch cultivations, the product EcFbFP is approximately increased fourfold compared to batch cultivations. The experiments on shake flask level showed the same biomass and product results as the cultivation with the stirred tank reactor, validating the comparibility of cultivation conditions between the membrane-based fed-batch shake flask and the stirred tank reactor.

## Conclusions

The membrane-based fed-batch shake flask is an innovative screening tool for small-scale cultivations. In the initial part of screening the fed-batch shake flask in combination with the RAMOS-device can be used to identify, amongst others, promising strains, process settings, and media compositions. A detailed analysis can then be conducted with parallelly run offline fed-batch shake flasks. When no online device is available, the offline fed-batch shake flask can be used as a stand-alone device to be sampled at pre-determined sampling time points. Simple in its set-up, yet versatile in its application, the membrane-based fed-batch shake flask offers the possibility to test in parallel a diverse number of fed-batch settings in an easy and reliable manner. The set-up variations of the reservoir were systematically analyzed for cultivations with the fast-growing organism *E. coli* and provide the basis for a quick adaptation to further biological systems of varying growth rates. Two main parameters to adapt the amount of released substrate, quantified in this study with the measurement of the oxygen transfer rate in the RAMOS device, are the initial substrate concentration in the reservoir and the membrane used for the controlled release. A good precognition of the biological system is of advantage, as this simplifies the choice of an appropriate membrane. In presence of cellulases for example, the membrane integrity of cellulosic dialysis membranes might be impaired. With the help of a newly developed membrane tip, the utilizable range of membranes is extendable from flexible dialysis membranes to stiff paper-like microfiltration membranes. For microbiological systems, which are sensitive to increasing osmolality values, the microfiltration membrane is a more suitable option as the concentration of the culture medium due to water diffusion to the reservoir can be reduced. The OTR level of the fed-batch phase can be varied to more than fivefold with the change of membranes and can be extended even further by altering the initial glucose concentration in the reservoir. Additionally, the variation of the membrane area and the MWCO offer further opportunities to fine-tune the substrate release as well as to influence batch peak characteristics.

In this work, modifications of the reservoir construction and optimized handling procedures are presented to ensure reproducible performance. The selected shaking parameters are sufficient to ensure a homogenous composition in the reservoir, despite the usage of high glucose concentrations in combination with narrow reservoir parts. Measurement of ammonium and phosphate in the culture broth and the reservoir with the dialysis membrane showed that the reservoir does not act as a permanent nutrient sink. These factors provide the basis to adequately imitate the feeding via a pump into a stirred tank reactor.

The applicability of the membrane-based fed-batch as a screening tool was demonstrated in a parallel cultivation with a laboratory-scale stirred tank reactor. The working volume of the shake flask cultivation and the stirred tank reactor cultivation was varied by a factor of 100. Equivalent biomass and product concentrations were obtained on both scales. The feeding via a pump and the controlled release from the reservoir system resulted in similar pH, osmolality and acetate values, validating comparative environmental conditions for cells in both types of bioreactors.

In future applications, the simultaneous release of substrate and pH-stabilizing components from the reservoir could reduce the amount of buffer necessary for shake flask cultivations. This would enable the realization of operation conditions even closer to stirred tank reactors with pH control. Further, the membrane-based fed-batch shake flask can also be used for downscaling. When an existing fermentation process needs to be optimized or an improved strain needs to be evaluated for its compatibility with the fermenter scale, the small-scale screening allows the testing of a larger number of possibly influencing parameters at much lower costs.

## Methods

### Strain

All cultivations were conducted with the model organism *E. coli* BL21 (DE3) pRhotHi-2-EcFbFP (GenBank Number: ABN71355) [14]. The production of a flavine mononucleotide binding fluorescence protein is under the control of a *lac* operon [53, 54]. Without induction, a fluorescence signal is detectable due to leaky expression [14, 55].

### Media

As complex medium LB medium was used for *E. coli* pre-cultures containing 10 g/L peptone from casein (Carl Roth, Karlsruhe/Germany), 5 g/L yeast powder extract for bacteriology (Carl Roth, Karlsruhe/Germany) and 5 g/L NaCl. The pH value was adjusted to pH 7.0–7.2. 50 mg/L kanamycin sulfate was added to maintain selection pressure.

All cultivation experiments of *E. coli* were performed in the modified Wilms-MOPS mineral medium [52, 56] with the base solution consisting of 6.98 g/L (NH_4_)_2_SO_4_, 3 g/L K_2_HPO_4_, 2 g/L Na_2_SO_4_, 41.85 g/L (*N*-morpholino)-propane sulfonic acid (MOPS). The pH value was adjusted to 7.5 with NaOH. Before each experiment, the base solution was supplemented with 0.5 g/L MgSO_4_·7H_2_O, 0.01 g/L thiamine hydrochloride, 1 mL/L trace element solution [0.54 mg/L ZnSO_4_·7H_2_O, 0.48 mg/L CuSO_4_·5H_2_O, 0.3 mg/L MnSO_4_·H_2_O, 0.54 mg/L CoCl_2_·6H_2_O, 41.76 mg/L FeCl_3_·6H_2_O, 1.98 mg/L CaCl_2_·2H_2_O, 33.4 mg/L Na_2_EDTA (Titriplex III)] and 50 mg/L kanamycin sulphate to maintain selection pressure.

The culture medium of batch cultivations was supplemented with a glucose solution to achieve a final concentration of 20 g/L glucose. In the culture medium of fed-batch cultivations, sterile distilled water was used to compensate the omitted glucose volume to maintain the same composition of the other medium components.

### Cultivation conditions

All cultivations were conducted with orbital shakers (LS-X, Kühner, Birsfelden/Switzerland) at 350 rpm, 50 mm shaking diameter at 37 °C. The RAMOS technology was used for online monitoring of the cultivation progress. The device is an in-house build apparatus. Commercial versions are available from Kühner AG (Biersfelden/Switzerland) or HiTec Zang GmbH (Herzogenrath/Germany). The oxygen transfer rate (OTR) was measured as a duplicate in the RAMOS device. Optionally offline batch and fed-batch shake flasks were used to provide samples for offline analysis.

For cryo cultures, cells were grown in complex medium until mid-exponential phase and then harvested. 750 μL of the culture was added to 250 μL of 80% (w/w) glycerol, used as cryo-protectant, homogenized and immediately frozen at −80 °C. For preculture cultivation, one cryo vial was resuspended in 11.5 mL complex medium. In the mid-exponential phase, the culture was harvested and centrifuged at 4000 rpm and 4 °C for 15 min. The cells were resuspended in the main culture medium and were used to inoculate the main culture at an OD_600_ of 0.5 if not otherwise mentioned. 10 mL culture volume was used for all main culture cultivations.

Parallel cultivations were conducted in the laboratory-scale stirred tank reactor and in the RAMOS device. For the batch cultivation, an Applikon Biotechnology fermenter ez-Control (Applikon Biotechnology B.V., Delft/Netherlands) was used with a dual six-blade Rushton impeller (45 cm diameter) and a working volume of 1 L. For fed-batch cultivations a Sartorius BIOSTAT^®^ Bplus fermenter (Sartorius, Göttingen/Germany) with a dual six-blade Rushton impeller (53 cm diameter) and a working volume of 1 L was used. For both stirred tank reactors, the temperature was controlled at 37 °C, and no active pH regulation was incorporated. A ring sparger was used for an aeration at 1 vvm. The dissolved oxygen tension was controlled at 30% air saturation by controlling the stirring rate. For the batch stirred tank reactor the rotation speed was varied between 500 and 1500 rpm and for the fed-batch stirred tank reactor between 500 and 800 rpm. In both batch and fed-batch operation mode, the same inoculated main culture was used respectively for RAMOS cultivations and stirred tank reactor cultivations. For the fed-batch stirred tank reactor, the glucose feed with a stock concentration of 250 g/L was accomplished with a pump (IPC RS232, Ismatec, Zürich/Switzerland) working at 4.2 mL/h. These settings were chosen to mimic the glucose release from the reservoir to the culture broth in a membrane-based fed-batch shake flask and were determined in a pre-experiment.

### Preparation of the membrane-based fed-batch shake flask

First, the required membrane tips were prepared. Washing and storage recommendations of the manufacturers were followed. The membranes used for cultivations are enlisted in Table 1. For flexible dialysis membranes, a circular membrane disc with the diameter of 14 mm was punched out and with the help of a silicone ring (inner diameter 4 mm, wall thickness 1 mm; Saint-Gobain, Charny/France) fixed on the front part of the membrane tip type A (Fig. 1b). An inhouse-built apparatus was used to ensure a crease-free and consistent spanning of the membrane. For stiff paper-like membranes, a circular membrane disc with the diameter of 7 mm was punched out and placed in the screw cap of the membrane tip type B (Fig. 1b). With the help of a gasket, the body of the membrane tip was screwed liquid-tight on the cap. To test for possible leakages, the assembled membrane tip was filled with liquid, preferably water to minimize alterations of the reservoir composition. The filled tip was positioned in a semi-micro cuvette and placed in a centrifuge. The centrifuge was operated at room temperature at 500 rpm for a minimum of 7 min. Only membrane tips that were liquid-free at the bottom of the cuvette were used further. The membrane tip was then loaded with a stainless-steel ring of 3.1 g and connected to the flexible tube (PharMed^®^ BPT, inner diameter 2.06 mm, wall thickness 0.8 mm; Saint-Gobain, Charny/France) of the reservoir. Silicon rubber rings (inner diameter 4 mm, wall thickness 1 mm; Saint-Gobain, Charny/France) were used to tighten both ends of the flexible tube with the respective connecting part, to the modified steel tube nozzle at the lower end of the glass tube or to the nozzle of the membrane tip. The modified steel tube nozzle, connected via UV bonding (UV curing at 366 nm, GB 368, Delo, Windach/Germany) with the glass tube, replaces the glass olive connection designed for the initial prototype [14]. This modification improved the conformity between reservoir systems of different shake flasks and increased their robustness towards functional stress. The assembled reservoir was then centrally placed in the shake flask and autoclaved. A previously prepared reservoir solution was sterile pipetted into the reservoir and gas bubbles entrapped through the filling procedure were removed with the help of a desiccator evacuated with a vacuum pump (Type 113042, LVS, Ilmenau/Germany). The evacuation and pressure normalization steps were repeated until no more gas bubbles ascended in the reservoir liquid. The culture broth was then filled sterile into the shake flask, and the device was placed on the shaker to start experimentation.

To correct offline data retrieved from the culture broth and from the reservoir due to concentration or dilution effects, the content of the shake flask and the reservoir were corrected with respective factors. The cell dry weight was corrected with a factor representing the ratio of the weight initially at the start and the weight at the sampling point of cultivation. A constant density for the culture broth was assumed. For the reservoir, a concentration-density correlation was used to convert the weight of the reservoir content to a volume. Offline samples taken from the reservoir were corrected with the factor obtained as a ratio of the initial volume and the volume at sampling time point. In this study, corrections were undertaken for CDW (g/L), phosphate (g) and ammonium (g) measurements. All other values depict the actually measured concentrations, in turn showing the current environmental conditions of cells.

To address the question whether a concentration gradient is prevalent along the reservoir height of the membrane-based fed-batch shake flask, samples were taken at the reservoir extremities to analyze the glucose concentrations via HPLC (Fig. 3). The reservoir was unscrewed from the flask with a minimal amount of jerk to reduce falsifications while sampling. Further, a clamp was placed on the flexible tube to avoid any intermixture while withdrawing fluid using a syringe at the respective sampling points. Samples were taken at the upper liquid surface, the middle portion of the glass tube and directly behind the membrane by piercing through the membrane from the front. For four different glucose concentrations from 250 to 750 g/L in the reservoir, the samples were taken for 72 h and analyzed.

Depending on the settings to be tested, the fed-batch reservoir contained a volume of 1–4 mL composed of 75–750 g/L initial glucose concentration and optionally the dye blue dextran (Sigma-Aldrich, Steinheim am Albuch/Germany) at 1–4 g/L. Blue dextran is a dye with a molecular weight of 2 million g/mol and, therefore, it is not able to pass the membrane without leakage. It can be traced in the supernatant using an absorbance measurement at 615 nm. In a study presented by Susanto et al. [57] evaluating the effect of adsorptive fouling of cellulosic membranes with dextrans, it was shown that no significant water flux reduction or alteration of membrane properties occurred. This was verified in this study in parallel cultivations with the reservoir containing blue dextran and omitting blue dextran. No significant difference in the progress of the OTR could be seen for cultivations without blue dextran in comparison to cultivations with 2 g/L blue dextran in the reservoir (see Additional file 3). No interference of blue dextran was observed for the variation range between 1 and 4 g/L (data not shown). In all experiments with offline samples, the dye blue dextran was added to the reservoir composition to achieve a final concentration of 4 g/L. For experiments with the variation of glucose concentration in the reservoir 1 g/L blue dextran was used. The usage of blue dextran was omitted in all other experiments.

### Offline sample analysis

The optical density (OD_600_) was determined from three samples in a standard 1 cm cuvette at 600 nm using a spectrophotometer Genesys 20 (Thermo Fisher Scientific, Bonn/Germany). The samples were diluted with 0.9% (w/v) sodium chloride solution to measure in the linear correlation range of 0.1 < OD_600_ < 0.3. 0.9% (w/v) sodium chloride solution was used as a blank. To determine cell dry weight (CDW), 5 mL of the respective culture broth sample was centrifuged in a previously dried and weighed reaction tube with 4000 rpm at 4 °C for 20 min. The resulting cell pellet was dried at 80 °C for a minimum of 48 h.

The pH value of the respective supernatant was measured using a pH 510 pH/mV/°C meter (Eutech Instruments Europe, Nijkerk/Netherlands). 50 µL supernatant from three samples were used for measuring the osmolality with the help of an Osmomat 030 (Gonotec, Berlin/Germany). Before measurement, a two-point calibration was conducted with water and calibration standards. The calibration standard (300, 500 or 850 mOsmol/kg, Gonotec, Berlin/Germany) closest to the expected osmolality was used for calibration. Ammonium and phosphate quantification of the supernatant were conducted according to the manual provided in the respective Spectroquant test kits (Merck KGaA, Darmstadt/Germany). The measurements were conducted in 1 cm cuvettes (Kartell, Noviglio/Italy) in the photometer Spektroquant NOVA 60 (Merck KGaA, Darmstadt/Germany).

The quantitative measurement of glucose and acetate was performed by means of high-performance liquid chromatography (Dionex HPLC Ultimate 3000, Sunnyvale/USA), equipped with an organic acid resin HPLC pre-column (40 × 8 mm) as well as an organic acid resin HPLC separation column (250 × 8 mm) (both from CS-Chromatography, Langerwehe/Germany). The supernatant of samples centrifuged for CDW determination was filtered using 0.2 μm cellulose acetate filters (VWR International, Darmstadt/Germany), diluted and stored at −20 °C until measurement. The eluent used was 5 mM sulphuric acid solution at a flow rate of 0.8 mL/min. A constant temperature of 60 °C was set during the measurement. The compounds were detected by measuring the refractive index using a Shodex RI-101 detector (Techlab, Erkerode/Germany). The Chromeleon software version 6.8 (Dionex Softron, Germering/Germany) was used for the subsequent analysis and peak calculation.

The fluorometric quantification of the fluorescence protein, FbFP, was determined by measuring four times 200 μL culture broth samples in black 96 well microtiter plates with clear bottoms (BD Falcon TC-treated Black/Clear, Corning Incorporated, Tewksbury/USA). The Synergy4 microtiter plate reader (Xenon flashlight, intensity 100, BioTek, Winooski/USA) was used to measure fluorescence at an excitation wavelength of 450 nm and an emission wavelength of 495 nm. In parallel, the absence of blue dextran in 200 μL supernatant was photometrically analyzed at 615 nm to ensure membrane integrity. The software Gen5 1.07 (BioTek, Winooski/USA) was applied for data collection.

## Additional files

**Additional file 1.** Effect of inoculation optical density variation on oxygen transfer rate in *E. coli* fed-batch cultivations (duplicates). Variation of inoculation OD_600_ between 0.3 and 1.5; cultivation conditions: Wilms-MOPS-mineral medium (0.2 M MOPS, 250 g/L glucose in the reservoir), temperature: 37 °C, shaking frequency: 350 rpm, shaking diameter: 50 mm, culture broth volume: 10 mL, reservoir filling volume: 2 mL, dialysis membrane: Reichelt (1) dialysis membrane, membrane area: 18.1 mm^2^; strain: *E. coli* BL21 (DE3) pRhotHi-2-EcFbFP.

**Additional file 2.** Effect of membrane thickness of a dialysis membrane on oxygen transfer rate in *E. coli* fed-batch cultivations (duplicates). Variation of membrane thickness between 28 µm (Reichelt (2) dialysis membrane) and 42 µm (Reichelt (1) dialysis membrane); cultivation conditions: Wilms-MOPS-mineral medium (0.2 M MOPS, 250 g/L glucose in the reservoir), temperature: 37 °C, shaking frequency: 350 rpm, shaking diameter: 50 mm, culture broth volume: 10 mL, inoculation OD_600_: 0.5, reservoir filling volume: 2 mL, membrane area: 18.1 mm^2^; strain: *E. coli* BL21 (DE3) pRhotHi-2-EcFbFP.

**Additional file 3.** Effect of the dye blue dextran in the reservoir on oxygen transfer rate in *E. coli* fed-batch cultivations (duplicates). **a** Duplicate cultivations with 8.6 mm^2^ membrane area **b** Duplicate cultivations with 18.1 mm^2^ membrane area; cultivation conditions: Wilms-MOPS-mineral medium (0.2 M MOPS, 250 g/L glucose in the reservoir), temperature: 37 °C, shaking frequency: 350 rpm, shaking diameter: 50 mm, culture broth volume: 10 mL, inoculation OD_600_: 0.5, reservoir filling volume: 2 mL, dialysis membranes: Reichelt (1) dialysis membrane; strain: *E. coli* BL21 (DE3) pRhotHi-2-EcFbFP.

## Abbreviations

CDW: cell dry weight
CTR: carbon dioxide transfer rate
*E. coli*: *Escherichia coli*
EcFbFP: *E. coli* codon bias-optimized FbFP
FbFP: flavin-mononucleotide-based fluorescent reporter protein
HPLC: high-performance liquid chromatography
MCE: mixed cellulose ester
MOPS: (*N*-morpholino)-propane sulfonic acid
MWCO: molecular weight cut-off
OD_600_: optical density at the wavelength of 600 nm
OTR: oxygen transfer rate
PC: polycarbonate
PES: polyether sulfone
PET: polyethylene terephthalate
RAMOS: respiration activity monitoring system
rpm: rotations per minute
RQ: respiratory quotient
